# Supplementary material for: Design, Synthesis, and Biological Evaluations of a Novel Resveratrol-Type Analogue Against VEGF
Source: Molecules. 2025 May 27;30(11):2345. doi: 10.3390/molecules30112345 (PMC12155980; doi:10.3390/molecules30112345)
Supplement: Supplementary file 1 [file molecules-30-02345-s001.zip › molecules-3659954-supplementary.pdf]

Design, Synthesis, and Biological Evaluations of a Novel Resveratrol-Type Analogue Against VEGF

Shengying Lin, Maggie Suisui Guo, Roy Wai-Lun Tang, Yutong Ye, Jiahui Wu, Yuen Man Ho, Ran Duan, Ka Wing Leung, Tina Ting-Xia Dong and Karl Wah-Keung Tsim *

Center for Chinese Medicine R & D, Division of Life Science, The Hong Kong University of Science and Technology, Clear Water Bay, Kowloon, Hong Kong, China; lishlin@ust.hk (S.L.); maggieguo@ust.hk (M.S.G.); roytwl@ust.hk (R.W.-L.T.); yyebl@connect.ust.hk (Y.Y.); jwuct@connect.ust.hk (J.W.); janetho@ust.hk (Y.M.H.); duanran@ust.hk (R.D.); lkwing@ust.hk (K.W.L.); botina@ust.hk (T.T.-X.D.)

* Correspondence: botsim@ust.hk; Tel.: +852-2358-7332

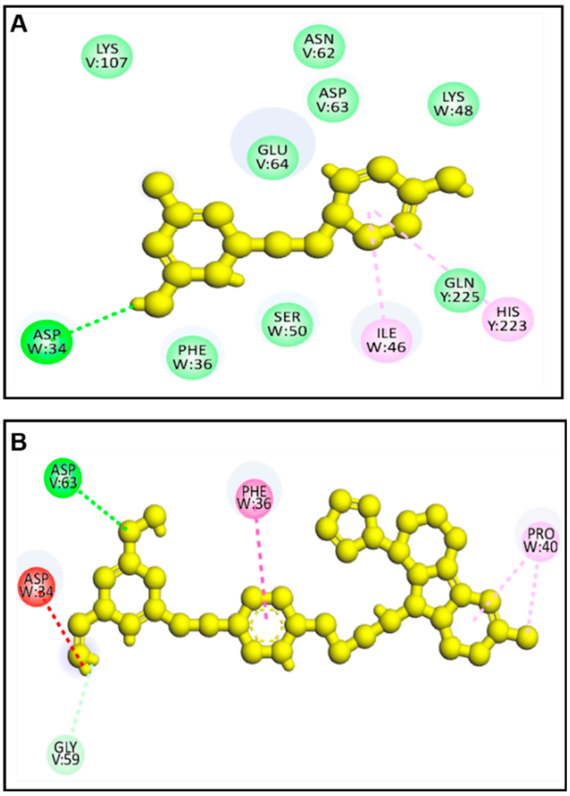

Figure S1. Protein-ligand interactions between VEGF and resveratrol (**A**) or RE-1 (**B**). Figures were generated from Discovery Studio 2024 (https://discover.3ds.com/, accessed on 20-01-2025).

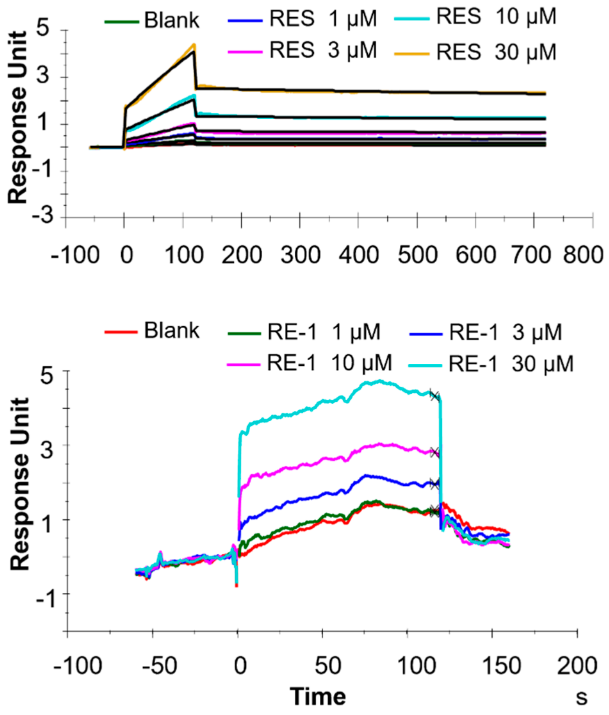

Figure S2. SPR binding of resveratrol and RE-1. Immobilized VEGF protein was placed onto a sensor chip, while analytes were flowing through the chip. RUs were recorded and calculated by GE Biacore T200 control software.

Table S1. Prediction of ADMET properties, including TPSA, Log P, intestinal absorption and so on, was conducted under Optibrium mode of SEESAR software (Version 13.0; https://www.biosolveit.de/, accessed on 11-04-2024). [a, b] following Lipinski's Rule of Five.

Analyte	MW (< 500 Da) [a]	TPSA (< 140 Å^2)	Log P (< 5) [b]	BBB (−)	CYPs Toxicity	HIA (+)	P-gp (−)
RES	228.2	60.7	2.97	−	Medium	+	−
RE-1	611.1	37.5	4.98	−	Medium	+	−
